# Acupoint application inhibits nerve growth factor and attenuates allergic inflammation in allergic rhinitis model rats

**DOI:** 10.1186/s12950-020-0236-9

**Published:** 2020-02-10

**Authors:** Wenzhan Tu, Xiaolong Chen, Qiaoyun Wu, Xinwang Ying, Rong He, Xinfa Lou, Guanhu Yang, Kecheng Zhou, Songhe Jiang

**Affiliations:** 1 Department of Physical Medicine and Rehabilitation, The Second Affiliated Hospital and Yuying Children’s Hospital of Wenzhou Medical University, Wenzhou, Zhejiang China; 2 Integrative & Optimized Medicine Research Center, China-USA Institute for Acupuncture and Rehabilitation, Wenzhou Medical University, 268 Xue Yuan Xi Road, Wenzhou City, Zhejiang 325027 People’s Republic of China

**Keywords:** Acupoint application, Allergic rhinitis, Nerve growth factor, Allergic inflammation, IL-4, Mast cells

## Abstract

**Background:**

Acupoint application therapy (AAT) has been widely used to treat allergic inflammation induced by allergic rhinitis (AR). The therapeutic effect of acupoint application is obvious. But the underlying therapeutic mechanism is still indistinct. Nerve growth factor (NGF) expression showed a dramatic rise in nasal mucosa tissue after AR, and allergic inflammation also increased significantly. To demonstrate how AAT can improve allergic inflammation by down-regulating the expression of NGF, AR rat models were established by intraperitoneal injection of ovalbumin (OVA) and nasal drops in SD rats. The number of nasal rubbing, sneezing and the degree of runny nose were observed and the symptoms were scored by behavioral symptom scoring method within 3 min. The expression levels of NGF and its downstream key proteins, such as IL-4, IL-5, IL-13, IgE and IFN-γ were determined by q-PCR, Western blot analysis, ELISA and immunofluorescence staining. Furthermore, H&E staining and toluidine blue staining were used to observe the pathological structure of nasal mucosa and mast cells in nasal mucosa, and the ultrastructure of nasal mucosa was observed by electron microscopy.

**Results:**

Our data demonstrated that acupoint application significantly reduced the score of behavioral symptoms, and decreased the expression levels of NGF and its downstream key proteins, including IL-4, IL-5, IL-13, IgE, as well as promoting the expression level of IFN-γ in nasal mucosa tissue in AR rats. Thus, the activation of IgE and viability of mast cells was inhibited.

**Conclusion:**

Our findings suggest that AAT can attenuate allergic inflammation by inhibiting the expression of NGF and its downstream pathway.

## Introduction

An increased incidence of allergic rhinitis (AR) has been manifested worldwide in recent years [1, 2]. AR, a chronic inflammatory disease of nasal mucosa, is mediated by immunoglobulin E (IgE) in T helper (TH) 2 type [3, 4]. AR, which stimulates the body by allergen, promotes the transformation of TH reaction from TH1 reaction to TH2 reaction evidenced by inhibiting the expression level of IFN-gamma secreted by TH1 cells, and promoting the expression levels of IL-4, IL-5 and IL-13 secreted by TH2 cells [5]. These cytokines attract mast cells, eosinophils and basophils to inflammatory sites. When the allergen stimulates the collective again, it binds to IgE on the surface of mast cells and produces an allergic reaction [6–8]. The main symptoms of AR were runny nose, itching and sneezing, seriously affecting patients’ quality of life and work [9–11]. Neurotrophins play an immunoregulatory role [12, 13], and mast cells and eosinophils can produce neurotrophins. Nerve growth factor (NGF) is an immune related protein mainly by enhancing the immune response of TH2 [14]. NGF binds to its high affinity receptor TRKA [15], TRPV-1, activates neuronal ion channels and promotes the production of VIP1, which can enhance TH2 immune response [16].

As a unique external treatment method of Traditional Chinese Medicine, acupoint application method can prevent and treat diseases by applying traditional Chinese medicine into acupoints, such as pills, powder and ointment, and using the dual effects of drug percutaneous absorption and meridian effect [17]. Compared with western medicine, acupoint application therapy (AAT) has less side effects and better curative effect [18]. Nevertheless, the specific mechanism of AAT in inhibiting allergic inflammation is complex, which may be related to inhibition of TH2 immune response [19]. NGF can enhance TH2 immune response [16]. In view of the correlation between the two, AAT may inhibit allergic inflammation by inhibiting the expression of NGF and its downstream proteins, such as IL-4, IL-5 and IL-13. To identify this hypothesis, AR rat models were established to observe the changes of inflammation and the expression of NGF and its downstream proteins. H&E staining and toluidine blue staining were used to observe the pathological structure of nasal mucosa and mast cells in nasal mucosa, and the ultrastructure of nasal mucosa was observed by electron microscopy before and after the treatment in order to evaluate the therapeutic effect of AAT on AR.

## Materials and methods

### Experimental animals

All male Sprague-Dawley rats (180~200 g) were bought from Wenzhou Medical University (Wenzhou, China). All rats were housed in a temperature-controlled (20~22 °C) room on a 12 h light/dark cycle at 45 ± 5% humidity and were given standard laboratory diet. Approval for the study was obtained from the Institutional Animal Care and Use Committee of Wenzhou Medical University. All SD rats were randomly divided into four groups: control group, model group, Prescription NO.1 acupoint application group, Prescription NO.2 acupoint application group.

### Ovalbumin (OVA) challenge protocol

Except the rats in control group, other SD rats were made into AR model. The rats in experimental groups were sensitized by intraperitoneal injection of 0.9% sodium chloride solution (1 mL) and OVA (0.3 mg) as antigen and aluminium hydroxide 30 mg as adjuvant once every other day for 8 times. Then they were sensitized by nasal drip containing 0.25% OVA and 0.9% sodium chloride solution once a day for 7 days. The control group was intraperitoneally injected with normal saline, nasal drip and atomization using the same method as above.

### Drug preparation and location of acupoints

Based on the agreement parties of the rehabilitation center of the Second Affiliated Hospital of Wenzhou Medical University for more than 20 years, the winter disease and summer treatment ointment was prepared. The ingredients of ointment were white mustard seed, asarum, Angelica dahurica, and Corydalis at 1:1:2:2. A total of 100 items of fine powder were studied. The ointment was prepared with ginger juice or honey, and sealed for preservation. The ointment in Prescription NO.1 acupoint application group was mixed with ginger juice, and in Prescription NO.2 acupoint application group, the ointment was mixed with honey. The acupoints applied were Dazhui, Fengmen (Shuang), Feishu (Shuang), Pishu (Shuang). *Point Location of Common Laboratory Animals* was used as a Reference Basis for Location. Dazhui acupoint is located between the 7th cervical vertebra and the 1st thoracic vertebra in the middle part of the back. The throttle is located between the ribs of the second thoracic vertebra. Feishu is located in the intercostal space under the third thoracic vertebra. Pishu is located in the intercostal space of the 12th thoracic vertebra.

### Behavioral tests

To observe the changes of nasal symptoms in SD rats, symptoms were observed and scored after sensitization (before AAT) and after 7, 14 and 28 days of the AAT. The specific method was to administrate 5 uL of 0.25% OVA and 0.9% sodium chloride solution nasal drip into each side. The number of nasal rubbing, sneezing and the degree of runny nose (within 3 min) were observed and the symptoms were scored. Specific scoring criteria are listed in Table 1 (*Chinese Medical Association Council of Otolaryngology, Editorial Committee of Chinese Journal of Otorhinolaryngology. Chinese standard of diagnosis and curative effect evaluation criteria of allergic rhinitis [J]. Chin J Otorhinolaryngol, 1998,33 (3): 134–135*). The allergic rhinitis model was considered successful when the total score was more than 5 points.

**Table 1:** Scoring criteria for nasal symptoms

Nasal symptoms	Scoring criteria	Score
Nasal itching	mild, rubbing the nose several times	1
	severe, scratching the nose, face endless	2
Continuous sneezing number	1 ~ 3	1
	4 ~ 10	2
	more than 11	3
Runny nose	running to the front nostril	1
	running over the front nostril	2
	running to all the face	3

### Acupoint application treatment

The application of acupoints was performed as mentioned above. Prescription No.1 was mixed with fresh Ginger Juice uniformly (1HG), and Prescription NO.2 was mixed with Honey uniformly (1HH). Rats in the control group (C group) were fed as usual. Rats in the model group (M group) were administrated 5 μl of OVA solution per nostril once a day. Rats in 1HG and 1HH groups were removed the hair on the back acupoints. In the 1HG group and 1HH group, isoflurane anesthesia was first used in rats, and then the ointment (0.5 cm × 0.5 cm) was applied at the acupoints for 1 h lasting for 28 days.

### Western blotting

After removing nasal mucosa tissue, the protein was homogenized and then determined by BCA kit (Thermo Fisher Science, Inc., Waltham, MA, USA). Protein (10 μl) was separated by 12% SDS PAGE and transferred to PDVF membrane. It was sealed at room temperature for 2 h with 5% skimmed milk and incubated with corresponding primary and secondary antibodies. Finally, the film (Thermo Fisher Science, Inc., Waltham, MA, USA) was detected by ECL system and photographed.

### Immunofluorescence staining

Nasal mucosa was fixed with 4% paraformaldehyde, and then dehydrated. After that, nasal mucosa was cut into sections (4 μm) following paraffin embedding. Slices were baked and dewaxed to water before incubated at room temperature with 3% hydrogen peroxide for 10 min. Subsequently, the slices were blocked in 10% goat serum and 0.3% Triton X-100 PBS solution for 1 h, followed by incubated overnight with corresponding antibodies (4 °C), and then IL-4 and IFN-gamma were double stained with mast cell markers. The primary antibodies and mast cell markers were rabbit anti-NGF (abcam, 1:100), rat anti-IL-4 (Santa, 1:200) and rabbit anti-Mast Cell Chymase (MCC, affinity, 1:100), rabbit anti-IFN-gamma (affinity, 1:100) and mouse anti-Mast Cell Tryptase (MCT, Santa, 1:200). The secondary antibody corresponds to the primary antibody. The nuclei were stained with DAPI. NGF, IL-4 and IFN-gamma positive cells in nasal mucosa sections were observed by fluorescence microscopy (FluoView FV1000, Olympus). Image Pro Plus 6 software (Media Cybernetics, Inc., Rockville, MD, USA) was used to analyze positive cells.

### Quantitative real-time PCR (RT-qPCR)

Total RNA was extracted from nasal mucosa using Trizol (Takara, Tokyo, Japan) reagent. Genes were synthesized according to the instructions of the Bio-Rad kit. Then 1 μl of DNA was taken for polymerase chain reaction (PCR). GADPH was used to normalize based on CT value. All the primers were designed and synthesized by Shanghai Jierui Bioengineering Co., Ltd. The sequences of primers are shown in Table 2.

**Table 2:** Primers for polymerase chain reaction

Primers	Sequence (5′-3′)
NGF	Forward: TTTGAGACCAAGTGCCGAGC
	Reverse: CACACACACGCAGGCTGTATCTAT
IL-4	Forward: CTTGCTGTCACCCTGTTCTGC
	Reverse: GTGGTGTTCCTTGTTGCCGT
IL-5	Forward: AGACGATGAGGCTTCCTGTTC
	Reverse: CTTCGCCACACTTCTCTTTTTG
IL-13	Forward: CCCTGACCAACATCTCCAGT
	Reverse: AGGTCCACAGCTGAGATGTC
IFN-γ	Forward: GAGGTGAACAACCCACAGATCCA
	Reverse: CGACTCCTTTTCCGCTTCCTTAG
IgE	Forward: CACTTCAAGGTTGCGGTCAA
	Reverse: GCTCATAACACACAGGGCAG
GADPH	Forward: GAGACAGCCGCATCTTCTTG
	Reverse: TGACTGTGCCGTTGAACTTG

### Enzyme-linked immunosorbent assay (ELISA)

The levels of NGF, IL-4, IL-5, IL-13, IFN-gamma and IgE in serum of rats were determined by enzyme-linked immunosorbent assay (ELISA). At room temperature, samples and diluents were added to the pore, and biotin antibodies were added to the pore. After washing, HRP and chromogenic solution of biotin were added. Finally, the terminating solution is added. The absorbance (OD) of each pore was measured at the wavelength of 450 nm.

### Hemotoxylin and eosin (H&E) staining and toluidine blue (TB) staining

#### H&E

Nasal mucosa was fixed with 4% paraformaldehyde, followed by dehydration and paraffin-embedding. After cut into sections (4 μm), the slices were baked before permeabilitization by xylene, dehydration by gradient alcohol, and dewaxing to water. Cells were stained by hematoxylin and eosin staining (Sigma Chemical Co.). Eosinophils were observed and counted. Double-blind and randomized analysis was performed.

#### TB

The pretreatment steps of the slices are as described above. The slices are stained with TB staining solution. Mast cells were observed and counted under a microscope. Double-blind and randomized analysis was performed.

### Statistical analysis

The original data were processed by SPSS 22.0 statistical software. The experimental results were expressed by X (+s) mean (+standard deviation), and all experimental data were analyzed by Graphpadprism 6 statistical software. LSD test was used for the comparison of homogeneous variance and Dunnett’T3 test for the comparison of uneven variance. Statistic with *P* value < 0.05 was considered as statistically significant.

## Results

### Effect of AAT on ovalbumin-induced rhinitis symptoms

**Fig. 1:**
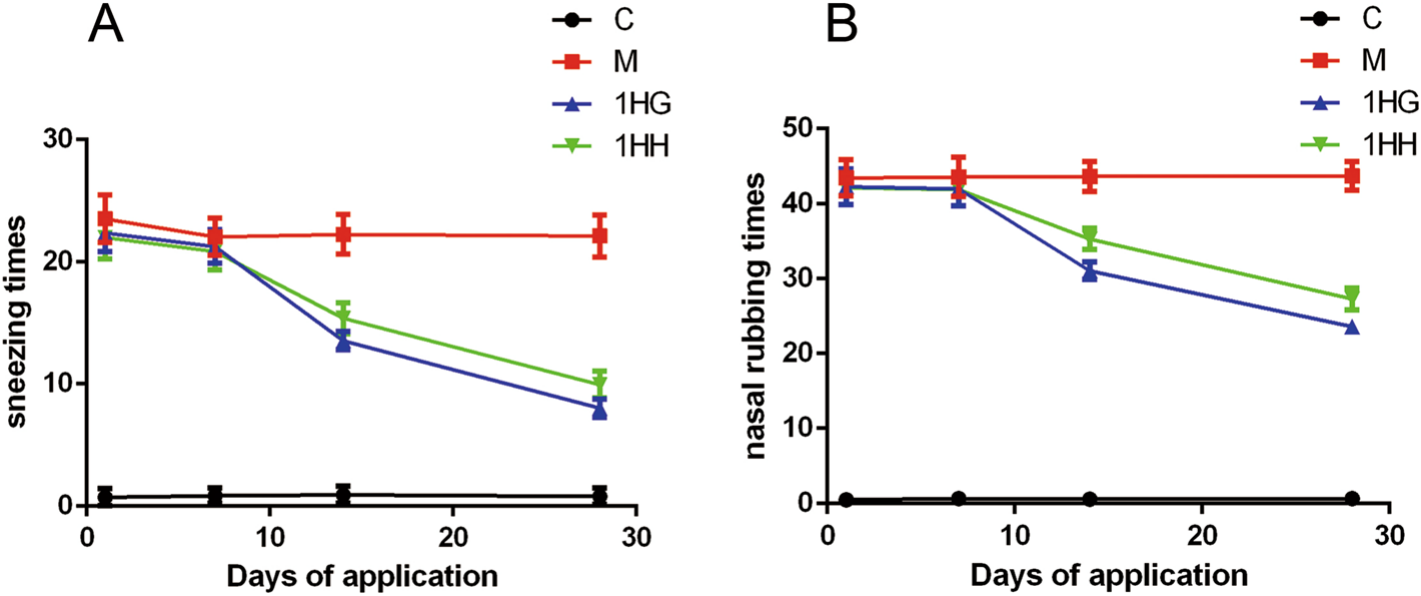
Effect of AAT on nasal symptoms in AR rats before and 7 days, 14 days and 28 days after acupoint application. Note: **a** The number of nose sneezes that occurred 10 min after OVA intranasal provocation; **b** The number of nose rubs that occurred 3 min after OVA intranasal provocation. The values represent the mean ± S.E.M (*n* = 10/group). Compared with M group, * *P* < 0.05, **P < 0.01,*** *P* < 0.001; compared with group 1HG, Significant differences # *P* < 0.05, ## *P* < 0.01, ### *P* < 0.001. AAT, Acupoint Application Therapy; AR, allergic rhinitis; OVA, ovalbumin; 1HH, prescription NO.2 mixed with Honey; 1HG, prescription NO.1 mixed with fresh Ginger Juice; M group, model group; C group, control group

The number of sneezing, rubbing nose and the degree of runny nose were scored and recorded before and after 7, 14 and 28 days of AAT. The results showed that there were no obvious symptoms of rats in control group, and there was no significant difference in the scores of rats in each group on the 7th day after AAT (*P* > 0.05). After 14 and 28 days of application, the behavioral scores of both 1HG and 1HH groups were significantly lower than those of M group (*P* < 0.01). Compared with the 1HG group, the behavioral score of 1HH group was significantly higher (*P* < 0.05) (Tables 3 and 4) (Fig. 1).

**Table 3:** The number of nose sneezes that occurred 10 min after OVA intranasal provocation in AR rats before and 7 days, 14 days and 28 days after acupoint application

Group	n	1st day	7th day	14th day	28th day
C	10	0.733 ± 0.704	0.867 ± 0.640	0.933 ± 0.704**	0.800 ± 0.676**
M	10	23.533 ± 1.960	22.067 ± 1.534	22.267 ± 1.624	22.133 ± 1.727
1HG	10	22.400 ± 1.549	21.267 ± 1.387	13.533 ± 0.743**	8.000 ± 0.756**
1HH	10	22.000 ± 1.773	20.867 ± 1.506	15.400 ± 1.298**#	15.400 ± 1.298**#

The values represent the mean ± S.E.M. Compared with M group, *, *P* < 0.05; **, *P* < 0.01; ***, *P* < 0.001; Compared with 1HG group, #, *P* < 0.05; ##, *P* < 0.01; ###, *P* < 0.001; *1HH* Prescription NO.2 mixed with Honey, *1HG* Prescription NO.1 mixed with fresh Ginger Juice, *M group* Model group, *C group* Control group, *OVA* Ovalbumin, *AR* Allergic rhinitis

**Table 4:** The number of nose rubs that occurred 3 min after OVA intranasal provocation in AR rats before and 7 days, 14 days and 28 days after acupoint application

Group	n	1st day	7th day	14th day	28th day
C	10	0.533 ± 0.516	0.667 ± 0.488	0.600 ± 0.507**	0.667 ± 0.617**
M	10	43.467 ± 2.446	43.600 ± 2.613	43.667 ± 2.024	43.733 ± 1.907
1HG	10	42.333 ± 2.380	42.000 ± 2.268	31.068 ± 1.163**	23.600 ± 0.910**
1HH	10	42.200 ± 2.274	41.933 ± 2.219	35.333 ± 1.447**#	27.333 ± 1.496**#

The values represent the mean ± S.E.M. Compared with M group, *, *P* < 0.05; **, *P* < 0.01; ***, *P* < 0.001; Compared with 1HG group, #, *P* < 0.05; ##, *P* < 0.01; ###, *P* < 0.001; *1HH* Prescription NO.2 mixed with Honey, *1HG* Prescription NO.1 mixed with fresh Ginger Juice, *M group* Model group, *C group* Control group, *OVA* Ovalbumin, *AR* Allergic rhinitis

### Detection of NGF and its downstream IL-4, IL-5, IL-13 and IFN-gamma in nasal mucosa by western blotting

**Fig. 2:**
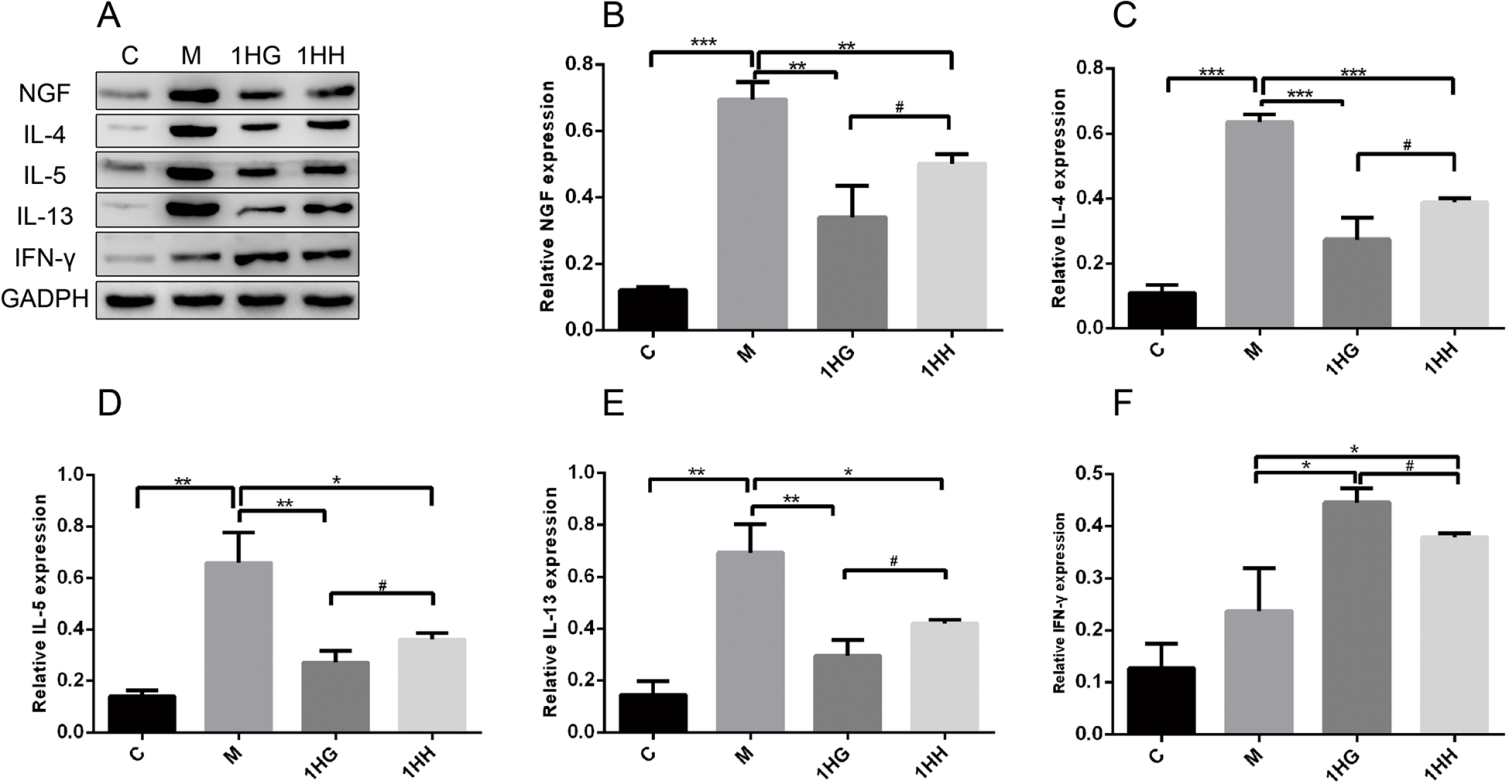
Effect of AAT on the expression of NGF, IL-4, IL-5, IL-13 and IFN-γ protein in nasal mucosa of AR rats. Note: **a** The protein level of NGF, IL-4, IL-5, IL-13 and IFN-γ was detected by western blot. **b** The protein level of NGF was detected by Western blot. **c** The protein level of IL-4 was detected by Western blot. **d** The protein level of IL-5 was detected by Western blot. **e** The protein level of IL-13 was detected by Western blot. **f** The protein level of IFN-γ was detected by Western blot. The values represent the mean ± S.E.M (*n* = 3/group). Compared with M group, *P < 0.05, **P < 0.01, ***P < 0.001; compared with 1HG group, Significant differences #*P* < 0.05, ## *P* < 0.01, ###*P* < 0.001; 1HH, prescription NO.2 mixed with Honey; 1HG, prescription NO.1 mixed with fresh Ginger Juice; M group, model group; C group, control group; AAT, Acupoint Application Therapy; AR, allergic rhinitis; NGF, nerve growth factor

Compared with the control group, the expressions of NGF, IL-4, IL-5 and IL-13 in nasal mucosa of the model group increased significantly (*P* < 0.001, *P* < 0.01), while the expression of IFN-gamma increased slightly and failed to achieve any significant difference (*P* > 0.05). Compared with the model group, the expression of NGF, IL-4, IL-5 and IL-13 in nasal mucosa of the 1HG and 1HH groups decreased (*P* < 0.001, *P* < 0.01, *P* < 0.05), while the expression of IFN-gamma increased (*P* < 0.05). Compared with the 1HG group, the expression of NGF, IL-4, IL-5 and IL-13 in nasal mucosa of rats in 1HH group increased significantly (*P* < 0.05), while the expression of IFN-gamma decreased significantly (*P* < 0.05) (Fig. 2).

### Expression of cytokines and mast cells were detected by immunofluorescence technique

**Fig. 3:**
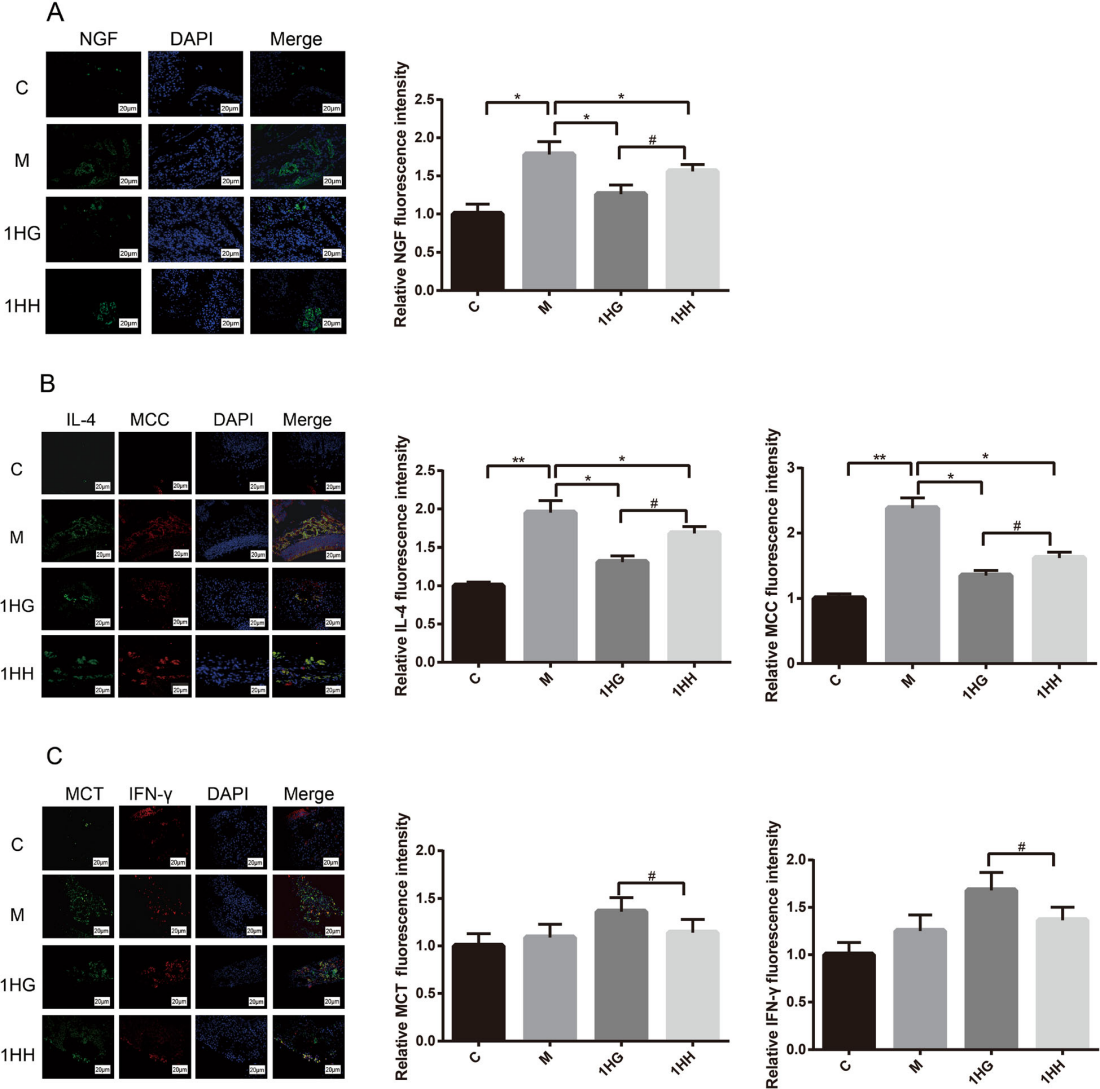
Effect of AAT on the expression of NGF、IL-4 and IFN-γ protein in nasal mucosa of AR rats. Note: **a** Effect of AAT on NGF protein expression in nasal mucosa with Olympus microscope. **b** The effects of Acupoint Application Therapy on mast cells localization of IL-4 with Olympus microscope. **c** The effects of Acupoint Application Therapy on mast cells localization of IFN-γ with Olympus microscope. The values represent the mean ± S.E.M (*n* = 3/group). Compared with M group, Significant differences *P < 0.05, **P < 0.01, ***P < 0.001; compared with 1HG group, Significant differences ^#^*P* < 0.05, ^##^*P* < 0.01, ^###^*P* < 0.001; AAT, Acupoint Application Therapy; AR, allergic rhinitis; NGF, nerve growth factor; 1HH, prescription NO.2 mixed with Honey; 1HG, prescription NO.1 mixed with fresh Ginger Juice; M group, model group; C group, control group

Compared with the control group, the positive spots of NGF and IL-4 protein in nasal mucosa of rats in model group increased significantly (*P* < 0.01, *P* < 0.05), but the positive spots of IFN-gamma protein did not increase significantly (*P* > 0.05). After 28 days of AAT, immunofluorescence results showed that the positive spots of NGF and IL-4 protein in nasal mucosa of rats in 1HG group and 1HH group significantly decreased compared with model group (*P* < 0.05). Compared with the 1HG group, the positive spots of NGF and IL-4 protein in nasal mucosa of 1HH group significantly increased (*P* < 0.05), while the positive spots of IFN-gamma protein significantly decreased (*P* < 0.05) (Fig. 3).

### Detection of cytokines and IgE gene expression in nasal mucosa by real-time fluorescence quantitative PCR

**Fig. 4:**
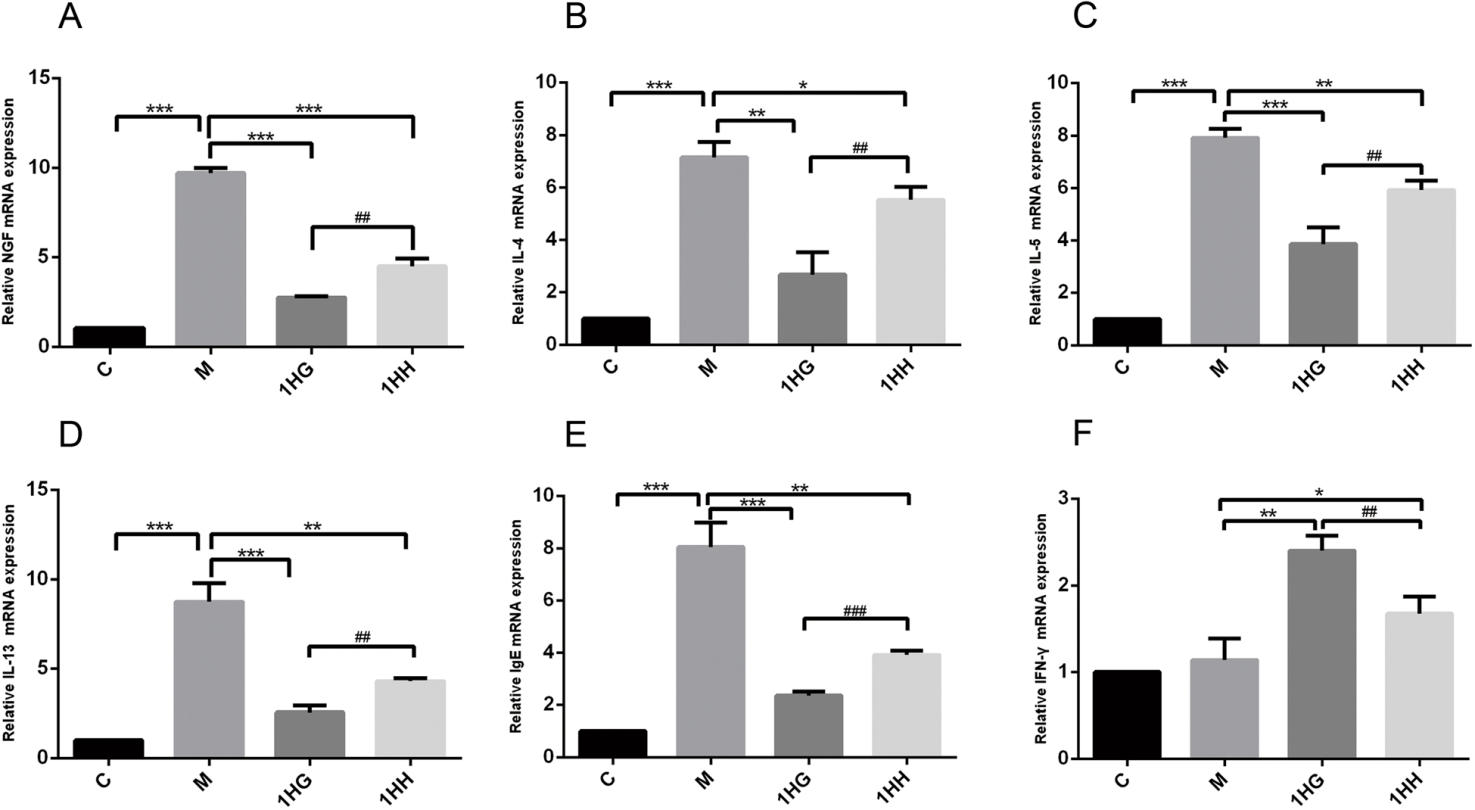
Effect of AAT on the expression of NGF, IL-4, IL-5, IL-13, IgE and IFN-γ mRNA in nasal mucosa of AR rats. Note: **a** The mRNA level of NGF was detected by real-time qPCR. **b** The mRNA level of IL-4 was detected by real-time qPCR. **c** The mRNA level of IL-5 was detected by real-time qPCR. **d** The mRNA level of IL-13 was detected by real-time qPCR. **e** The mRNA level of IgE was detected by real-time qPCR. **f** The mRNA level of IFN-γ was detected by real-time qPCR. The values represent the mean ± S.E.M (*n* = 3/group). Compared with M group, Significant differences *P < 0.05, **P < 0.01, ***P < 0.001; compared with 1HG group, Significant differences #*P* < 0.05, ##*P* < 0.01, ###*P* < 0.001; AAT, Acupoint Application Therapy; AR, allergic rhinitis; NGF, nerve growth factor; qPCR, quantitative PCR; 1HH, prescription NO.2 mixed with Honey; 1HG, prescription NO.1 mixed with fresh Ginger Juice; M group, model group; C group, control group

Compared with the control group, the expression of NGF, IL-4, IL-5, IL-13 and IgE mRNA in the model group increased (*P* < 0.001), while the expression of IFN-gamma mRNA increased slightly (*P* > 0.05). Compared with the model group, the expression of NGF, IL-4, IL-5, IL-13 and IgE mRNA in the nasal mucosa of rats in 1HG and 1HH groups decreased (*P* < 0.001, *P* < 0.01, *P* < 0.05), while the expression of IFN-gamma mRNA increased (*P* < 0.01, *P* < 0.05). Compared with the 1HG group, the expression of NGF, IL-4, IL-5, IL-13 and IgE mRNA in nasal mucosa of rats in 1HH group increased significantly (*P* < 0.001, *P* < 0.01), while the expression of IFN-gamma mRNA decreased significantly (*P* < 0.01) (Fig. 4).

### The serum levels of cytokines and IgE in AR rats were detected by ELISA

**Fig. 5:**
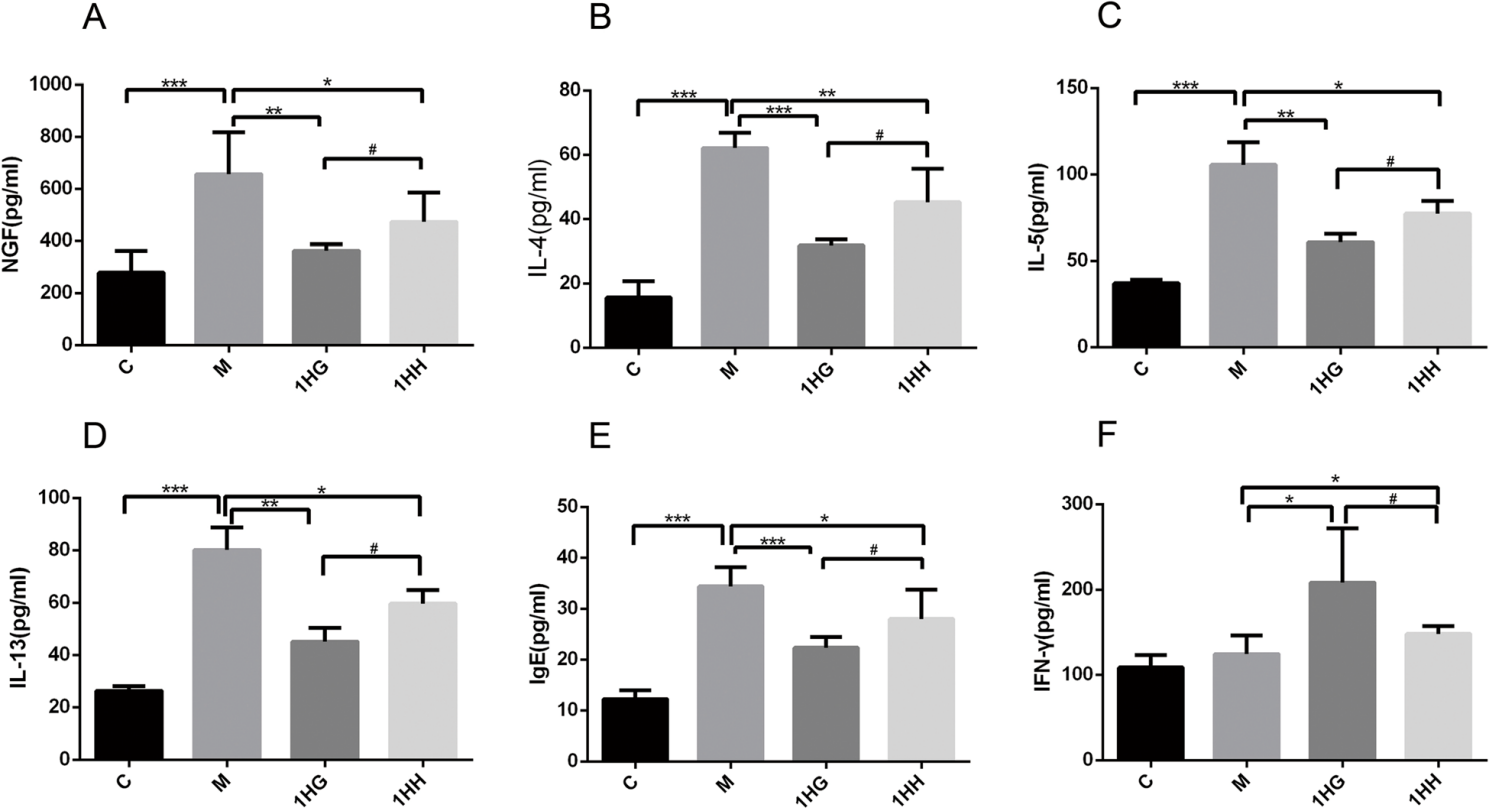
Effect of AAT on the expression of NGF, IL-4, IL-5, IL-13, IgE and IFN-γ in serum of AR rats. Note: **a** The serum level of NGF was detected by enzyme-linked immunosorbent assay (ELISA). **b** The serum level of IL-4 was detected by ELISA. **c** The serum level of IL-5 was detected by ELISA. **d** The serum level of IL-13 was detected by ELISA. **e** The serum level of IgE was detected by ELISA. **f** The serum level of IFN-γ was detected by ELISA. The values represent the mean ± S.E.M (*n* = 3/group). Compared with M group, Significant differences *P < 0.05, **P < 0.01, ***P < 0.001; compared with 1HG group, Significant differences #*P* < 0.05, ##*P* < 0.01, ###*P* < 0.001; AAT, Acupoint Application Therapy; NGF, nerve growth factor; ELISA, enzyme-linked immuno sorbent assay; 1HH, prescription NO.2 mixed with Honey; 1HG, prescription NO.1 mixed with fresh Ginger Juice; M group, model group; C group, control group

Compared with the control group, the serum levels of NGF, IL-4, IL-5, IL-13 and IgE in the model group increased (*P* < 0.001), and the expression of IFN-gamma increased slightly (*P* > 0.05). Compared with the model group, the serum levels of NGF, IL-4, IL-5, IL-13 and IgE in the 1HG group and 1HH group decreased (*P* < 0.001, *P* < 0.01, *P* < 0.05), while the expression of IFN-gamma increased (*P* < 0.05). Compared with the 1HG group, the serum levels of NGF, IL-4, IL-5, IL-13 and IgE in 1HH group were significantly increased (*P* < 0.05), while the expression of IFN-gamma was decreased significantly (*P* < 0.05) (Fig. 5).

### Pathological changes of nasal mucosa by H&E staining and the number and morphological changes of mast cells in nasal mucosa by TB staining

#### H&E staining

No obvious pathological changes were found in nasal mucosa of the control group. In model group, hyperemia, edema, gland hyperplasia, mucus secretion, tissue structure disorder and inflammatory cell infiltration appeared in nasal mucosa. After 28 days of AAT, the pathological degree of 1HG group and 1HH group were significantly reduced, and inflammatory cell infiltration was reduced. The pathological improvement degree in 1HG group was more obvious, and the number of inflammatory cells in nasal mucosa was the less.

#### TB staining

**Fig. 6:**
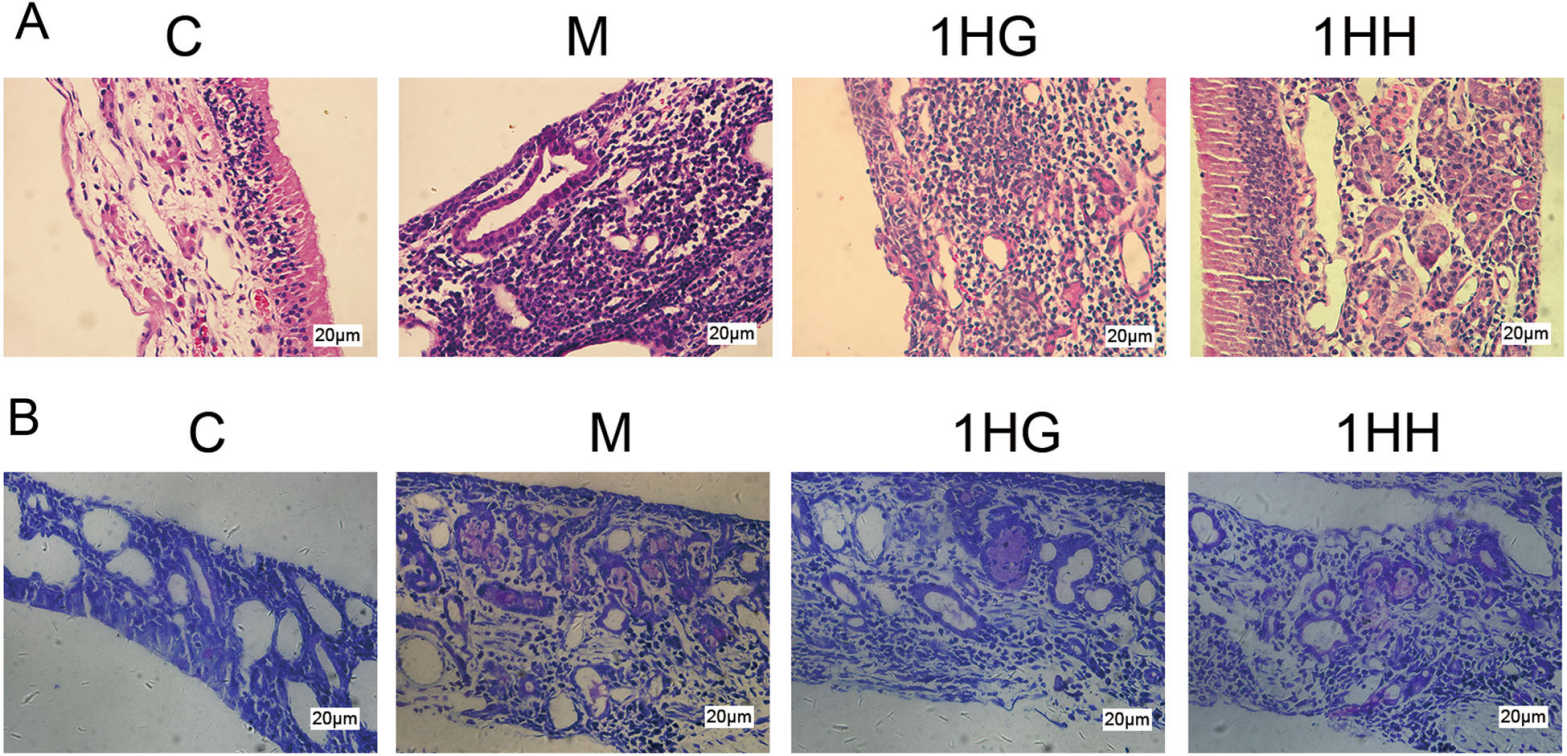
Effect of AAT on the proliferation of eosinophils and mast cells in nasal mucosa of AR rats Note: **a** Nasal mucosa tissue was stained with H&E for eosinophils. Original magnification 400×, scale bar = 20 μm. **b** Nasal mucosa tissue was stained with TB for mast cells. Original magnification 400×, scale bar = 20 μm. The values represent the mean ± S.E.M (*n* = 3/group). Compared with M group, Significant differences *P < 0.05, **P < 0.01, ***P < 0.001; compared with 1HG group, Significant differences #*P* < 0.05, ##*P* < 0.01, ###*P* < 0.001; AAT, Acupoint Application Therapy; H&E,hematoxylin and eosin; TB, toluidine blue; 1HH, prescription NO.2 mixed with Honey; 1HG, prescription NO.1mixed with fresh Ginger Juice; M group, model group; C group, control group

Mast cells were not found in the nasal mucosa of the control group, but a large number of purple-red mast cells were observed in the model group. After 28 days of treatment, the number of mast cells in both 1HH and 1HG groups decreased, especially in 1HG group (Fig. 6).

## Discussion

In this paper, we demonstrated that AAT can alleviate allergic inflammation in rats with AR, and inhibit IgE allergic reaction by inhibiting the activation of NGF and reducing the production of inflammatory factors (such as IL-4, IL-5, etc.). These results reveal the mechanism of AAT in the treatment of AR. A previous study reported that the occurrence of AR is closely related to the activation of NGF and neuroimmunity [20].

The common pathogenesis of AR is the imbalance of Th1/Th2 cell immunity. The main manifestation of AR is inflammatory disease with Th2 reaction [21]. Th2 cells mainly secrete cytokines such as IL-4, IL-5 and IL-13, which mainly stimulate humoral immune response. Among those cytokines, IL-4 most closely associated with AR [22, 23]. TH1 cells mainly secrete IFN-gamma and mediate cellular immune response. IFN-gamma closely related to AR [24]. The cytokines secreted by TH2 cells can promote the production of IgE (by B cells). IgE binds to high affinity receptor Fc receptor (Fc epsilon RI) (appeared in mast cells and basophilic granulocyte surface) [25], which promotes the degranulation of mast cells [26], and induces not only the release of histamine, leukotriene and other bioactive mediators, but a variety of pathological reactions (such as increased secretion of smooth muscle contraction and glandular mucus, dilatation of capillaries and small vessels, and increased permeability of blood vessels), leading to the occurrence of AR [27]. From the behavioral point of view, the symptoms of AR rats are consistent with the pathological reaction. After acupoint application, the symptoms of AR rats were alleviated slightly or significantly, indicating that acupoint application is effective for AR treatment.

NGF also participates in this immune response. NGF not only maintains the survival of neurons [28], but also plays an extensive role in immune, reproductive and hematopoietic systems [29–31], and directly participates in the immune response [32]. It has been reported that the cells involved in immune response, such as eosinophils, CD4 + T cells, some B cells and mast cells, can also produce and release NGF [33, 34]. There are a large number of sensory nerve fibers in nasal epithelial cells, most of which are markers of nociceptors, including trpv-1 and trpa-1 [35]. TH2 cytokines, such as IL-5, can activate lung noxious neurons and secrete VIP, thus stimulating lymphocyte to produce airway allergic reaction. These lymphocytes can secrete cytokines such as IL-5 and aggregate into effectors. Immune cells enter the disease state and further activate the sensory organs [36]. NGF promotes the proliferation and accumulation of immune cells through the aforementioned activation channels, and induces immune cells to release various bioactive factors (such as IL-4, IL-13, IL-5, IFN-gamma), which leads to the aggravation of allergic inflammation. In addition, immune cells such as eosinophils, mast cells and basophils can synthesize large amounts of NGF and further release [37], thus forming a positive feedback immune response, aggravating the progress of allergic rhinitis.

From the results of Western blot and PCR in this study, AAT can inhibit the mRNA and protein expression levels of NGF, IL-4, IL-5, IL-13, and promote the protein and mRNA expression of IFN-gamma. These results suggested that AAT can inhibit the formation of NGF in nasal mucosa and the positive feedback loop induced by NGF, while reduce the levels of IL-4 and NGF in nasal mucosa through nerve immune pathway of NGF/IL-4, and improve allergic inflammation in AR rats. In addition, our results suggest that IL-4 and IFN-gamma are co-expressed with mast cells. Compared with the control group, the co-expression of IL-4 and mast cell marker (chymase) [38] in nasal mucosa of AR rats increased. The up-regulation of chymase is related to the activation of mast cells. The results showed that mast cells were activated after AR. After acupoint application, the co-expression of IL-4 and chymase in nasal mucosa was lower than that in AR rats, and the co-expression of IFN-gamma and tryptase [39] was significantly higher than that in model group. Therefore, AAT can alleviate allergic inflammation in AR rats by inhibiting the expression of IL-4 in mast cells of nasal mucosa and increasing the expression of IFN-gamma in mast cells.

The powder is composed of four traditional Chinese medicine drugs, white mustard seed, asarum, Angelica dahurica and corydalis. White mustard seed has anti-inflammatory effect [40]. Angelica dahurica has anti-inflammatory effect by inhibiting mast cell degranulation and histamine release [41]. Asarum has anti-immune and anti-allergic effects. Compared with honey group, ginger group had stronger stimulation intensity, less volatilization of local water, and water content in skin increased sharply, which enhanced aseptic inflammation of local skin and made drugs more easily absorbed [42]. Therefore, compared with 1HH group, the symptoms in 1HG group were more obvious, which could inhibit the production of NGF, IL-4, IL-5, IL-13 and promote the production of IFN-gamma.

## Conclusion

Acupoint application therapy can improve the allergic inflammation response of AR rats by reducing the expression of NGF, IL-4, IL-13, IL-5 and IgE in nasal mucosa of AR rats and enhancing the expression of IFN-gamma. Moreover, ginger has a stronger therapeutic effect than honey.

## Data Availability

The datasets used and/or analyzed during the current study are available from the corresponding author on reasonable request.

## Abbreviations

AA: Acupoint application
AR: Allergic rhinitis
ELISA: Enzyme-linked ImmunoSorbent Assay
HE: Hematoxylin-Eosinstaining
IFN-gama: Interferon-gama
IgE: Immunoglobulin E
IL-13: Interleukin-13
IL-4: Interleukin-4
IL-5: Interleukin-5
NGF: Nerve growth factor
OVA: Ovalbumin
PBS: Phosphate buffered saline
PCR: Polymerase chain reaction
PFA: Polymerisatum formaldehydum
PVDF: Polyvinylidenedifluoride
SD: Standard deviation
SDS: Sodiumdodecyl sulfate
